# The complete mitochondria genome of *Calliphora vomitoria* (Diptera: Calliphoridae)

**DOI:** 10.1080/23802359.2016.1159930

**Published:** 2016-06-20

**Authors:** Lipin Ren, Qiyuan Guo, Weitao Yan, Yadong Guo, Yanjun Ding

**Affiliations:** a Department of Forensic Science, School of Basic Medical Sciences, Central South University, Changsha, Hunan, China;; b Engineering College, Yan Bian University, Yanji, Jilin, China

**Keywords:** *Calliphora vomitoria*, mitochondria genome, phylogenetic analyses, species identification

## Abstract

*Calliphora vomitoria* is a significant insect which belongs to the Calliphoridae family. In this study, the mitochondrial genome of *C. vomitoria* was completely sequenced for species identification. The entire mitogenome was 16,134 bp in length, composing of 13 protein-encoding genes, 22 transfer RNA genes and two ribosomal RNA genes, and then the array of the genes was similar to the other insects have discovered. The overall base compositions of A, G, C and T were 39.40%, 9.37%, 13.08% and 37.13% respectively. What is more, phylogenetic analyses tree indicated that entire mitochondria genome sequences of *C. vomitoria* had high degree of identification among the species listed in. We hope that the results from the present study will provide useful dipteran mitochondrial genomes information for the further studies on genetic structure and phylogenetic analyses of *C. vomitoria* in the species identifications.

Postmortem interval (PMI) time estimation has been pivotal and difficult in forensic medicine, especially in decayed cases (Yan et al. 2014). *Calliphora vomitoria* (*Linnaeus* 1758) belonging to the Calliphora genus, Calliphoridae family and Diptera order, is widespread in China except Hainan province. And the *C. vomitoria* is the first wave of sarcosaphagous flies to arrive and oviposit into animal carcasses in Europe (Senta Niederegger et al. 2013). Species identification of *C. vomitoria* is a crucial step in the estimation of the PMI. Viewing it as a potential marker in forensic entomology, we present the entire mitochondrial genome sequence of *C. vomitoria* for species identification and phylogenetic analysis (GenBank accession no. KT444440). The DNA sample received a unique identification code and is permanently stored in the Guo lab.

The *C. vomitoria* samples were collected in July 2014 from Changsha, China (28^°^9′N; 112^°^54′E). The complete genome was amplified in eight fragments (Nelson et al. 2012), and the double-stranded sequence was verified by prime walking within each long polymerase chain reaction (PCR) product. The entire fragments were amplified by TaKaRa MightyAmp Taq (Takara, Dalian, China) and performed on an Eppendorf Mastercycler gradient (Eppendorf, Hamburg, Germany) in 20 μL reaction volume. DNA fragments were sequenced on both strands by means of Sanger dideoxy sequencing method by the commercial service (Transduction Bio Co. Ltd. Wuhan, China). The complete genome of *C. vomitoria* was length of 16,134 bp including 13 protein coding genes, 22 transfer RNA genes, two ribosomal RNA genes and a control region as in other insects. The 1319 bp misc_feature region of *C. vomitoria* was located between 12S rRNA and *tRNA-Ile*. The genome was specifically AT biased, with 76.53% of the nucleotides being either A or T, which was slightly more AT-rich than that for *S. similis* (76.37%) (Yan et al. 2016) and *S. africa* (75.74%) (Fu et al. 2016), 12 of the 13 protein coding genes were identified with ATN as start codon coding for M except COI which is the equal to the former result (Zhong et al. 2016a,b).

Phylogenetic analysis was constituted by the 12 complete mitochondria gene sequences from six species of Calliphoridae, along with 2 species from Sarcophagidae family as outgroup species. Two of the Calliphoridae species, *L. cuprina and L. sericata* including multiple specimens, allow genomic variability within these species to be assessed. The sequence data from GenBank which are *L. porphyrina, C. vomitoria, C. rufifacies, H. ligurriens,* four *L. cuprina*, two *L. sericata* of Calliphoridae and *S. africa, S. similis* from Sarcophagidae family. Except *C. vomitoria*, the other Calliphoridae genomes originally published in Nelson’s paper (Nelson et al. 2012), *S. similis* published in mitochondrial DNA (Yan et al. 2016b) and *S. africa* published in mitochondrial DNA (Fu et al. 2016).

The phylogeny of Calliphoridae flies based on the complete mitochondria gene sequences was separated into several genetic clades (Figure 1). As an outgroup, the two Sarcophagidae samples were crowd together and clearly divided into the Calliphoridaid mitotypes. The monophyletic branches of the phylogenetic tree indicated that the composition of the *C. vomitoria* mitochondrial genome, particularly the gene lengths was much similar to that of another Calliphoridae fly, *L. sericata*, but the interspecific variations between them was 7.0%, which could distinguish these two species explicitly. The interspecific and intraspecific percentage genetic divergences were calculated. All values for maximum intraspecific variations of the Calliphoridae species were no more than 2%. The interspecific variations between species were larger than 5% except for *L. cuprina* and *L. sericata* which were no more than 2%, the results was consistent with the previous study (Nelson et al. 2012), which indicated that phylogenetic analysis of whole mt genome sequences resulted in much stronger support for discrimination between *C. vomitoria* and other five species, but week for discrimination between the *L. cuprina* and *L. sericata.*

**Figure 1.**
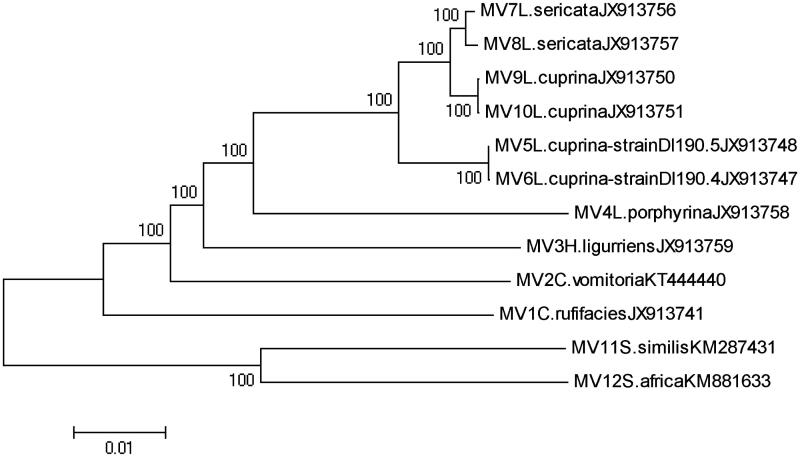
Neighbour-joining (NJ) tree of maximum synthetic-likelihood method for the entire mitochondria gene sequences from 6 species of Calliphoridae. Voucher ID, morphological species identification, and accession number are given in specimen label. Numbers on branches demonstrate the support value. Two from family Sarcophagidae are contained as an outgroup. Evolutionary distance divergence scale bar is 0.01.

Species identification of Sarcosaphagous flies by means of morphological methods is a difficult task, in particular for spawn and larva. Forensic scientists have to wait for the adult emergence (Wang et al. 2002). Now species identification techniques of molecular biology including molecular taxonomy (Smith & Baker 2008) serve as an effective supplement for morphological identification. The first provided complete genome of mitochondrial DNA of *C. vomitoria* which can be valuable for the implementation of the Calliphoridae database and species identification.
